# Tremor Artifact Presenting as Pseudo-Ventricular Tachycardia

**DOI:** 10.1016/j.jaccas.2026.108029

**Published:** 2026-06-03

**Authors:** Ata Ul Haiy, Muhammad Abdul Basit, Hasan Mahmood Mirza, Aaima Wasim, Ahmad Jalil, Ata Bajwa

**Affiliations:** a Department of Internal Medicine, United Health Services Wilson Medical Center, Johnson City, New York, USA; b Department of Cardiology, United Health Services Wilson Medical Center, Johnson City, New York, USA; c Department of Internal Medicine, King Edward Medical University, Lahore, Pakistan; d Department of Internal Medicine, Baptist Memorial Hospital, Oxford, Mississippi, USA; e Department of Electrophysiology, United Health Services Wilson Medical Center, Johnson City, New York, USA

**Keywords:** ECG artifact, pseudo-ventricular tachycardia, tremor artifact

## Abstract

**Background:**

Electrocardiographic artifacts can present as pseudo-arrhythmia, such as ventricular tachycardia (VT), leading to unnecessary interventions.

**Case Summary:**

A 47-year-old woman with hypertension, asthma, obstructive sleep apnea, lupus/Sjögren disease, and schizoaffective disorder was admitted for failure to thrive. Telemetry suggested polymorphic and monomorphic VT, prompting a rapid response. She was asymptomatic and hemodynamically stable, with a pulse 93/min. A baseline tremor was noted. Post–rapid-response electrocardiogram revealed an normal sinus rhythm with a QTc interval of 460 milliseconds. Preserved QRS complexes were seen to be embedded within the observed VT on telemetry review. Identification of the artifact pattern prevented further unwarranted interventions.

**Discussion:**

Tremor-induced artifacts can present as compelling VT mimics. Stable hemodynamics, concealed QRS complexes, and bedside examination are crucial in distinguishing pseudo-arrhythmia from true arrhythmia.

**Take-Home Messages:**

Electrocardiographic artifacts should be considered in clinically stable patients with telemetry VT alarms. Careful holistic approach to such alarms can avoid unnecessary invasive procedures and antiarrhythmic use.

## Visual Summary

**Figure:**
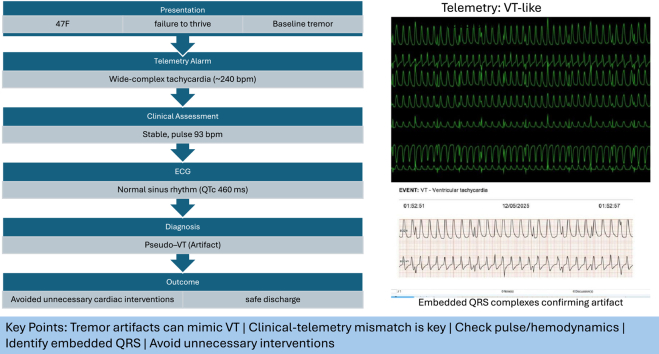

**Visual Summary:**
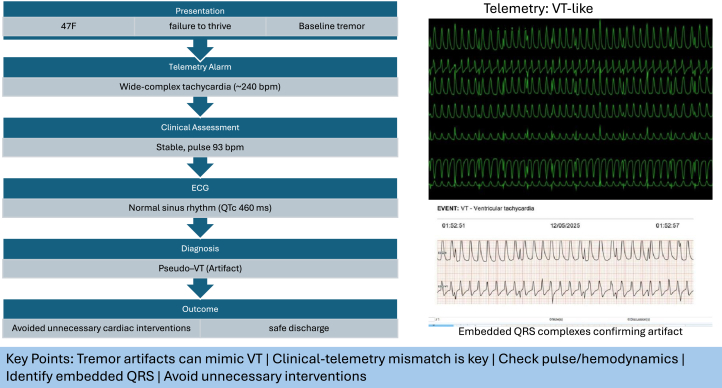
Tremor Induced Telemetry Artifact Mimicking Ventricular Tachycardia ECG = electrocardiogram; VT = ventricular tachycardia.

## Background

Ventricular tachycardia (VT) is defined as an arrhythmia consisting of at least 3 consecutive wide QRS complexes at a rate >100 beats/min.^1^ VT is broadly classified as monomorphic or polymorphic based on electrocardiography morphology. Monomorphic VT typically reflects a stable reentrant circuit, most often associated with myocardial scar, whereas polymorphic VT is characterized by beat-to-beat variability in the QRS morphology, which is often associated with myocardial ischemia, QT prolongation, and/or metabolic disturbances. Timely and accurate recognition of VT is crucial because delayed or inappropriate management may lead to significant morbidity and mortality.^1^

**Take-Home Messages**

• Tremor-induced telemetry artifacts can mimic ventricular tachycardia.
• Bedside clinical correlation and identification of embedded preserved rhythm can avoid unnecessary interventions.

Telemetry and 12-lead-electrocardiography are important tools for the detection and diagnosis of ventricular arrhythmia in hospitalized patients. Set electrocardiographic (ECG) criteria, including the Brugada algorithm, which describes the distinction between VT and supraventricular tachycardia with aberrancy, are beneficial to the diagnosis.^2^^,^^3^ However, telemetry is naturally vulnerable to artifacts brought on by patient movement, tremors, inadequate lead contact, or incorrect lead placement because ECG signals are captured through the chest wall and inadvertent muscular/motion signals might be picked up. Motion and tremor can therefore also be incorrectly considered as malignant ventricular arrhythmia including monomorphic and polymorphic VT and can lead to an escalation of treatment; however, this may not necessarily be clinically unstable.^4^ Misinterpretation can lead to invasive diagnostic procedures, extended hospital stays, and antiarrhythmic drug administration. Therefore, it is necessary to carefully correlate telemetry results with bedside clinical assessment, including assessment of hemodynamic stability and bedside pulse examination to distinguish true VT from artifact.^5^

Nevertheless, despite all interventions and structured diagnostic criteria, ECG artifacts still pose diagnostic challenges during routine clinical practice. We present a case of tremor-induced ECG artifact masquerading as VT, highlighting the importance of clinical correlation in telemetry interpretation and the prevention of unnecessary cardiac interventions.

## Case Presentation

A 47-year-old woman with hypertension, asthma, obstructive sleep apnea, systemic lupus erythematosus/Sjögren syndrome treated with hydroxychloroquine, and schizoaffective disorder treated with clozapine was admitted for functional decline and failure to thrive. She was noted to have tremors at baseline. During hospitalization, psychiatry diagnosed her with clozapine-induced catatonia. The patient was placed on telemetry monitoring for electrolyte abnormalities.

**Figure 1:**
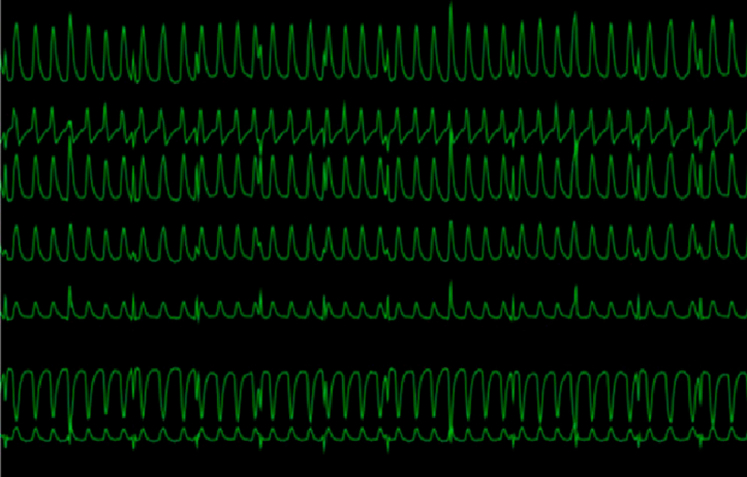
Telemetry Tracing of Pseudo-Ventricular Tachycardia

**Figure 2:**
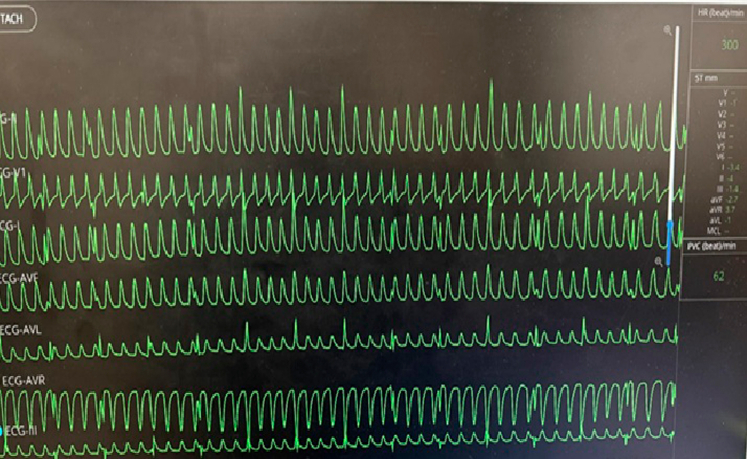
Telemetry Tracing Showing Pseudo-Ventricular Tachycardia

**Figure 3:**
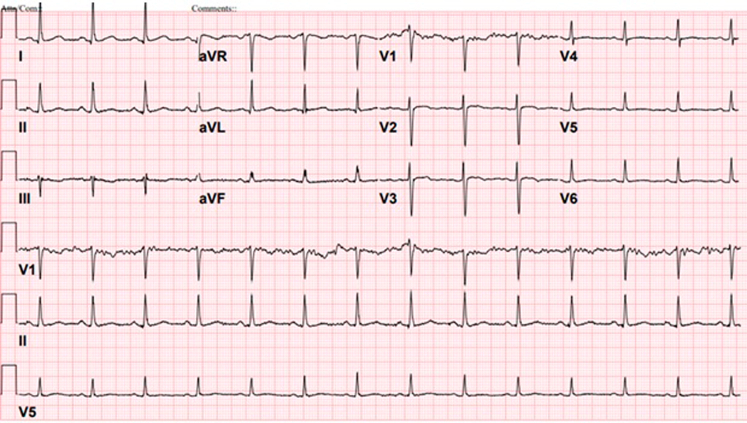
Electrocardiographic Tracing Done After Rapid Response

A rapid response was called on hospital day 3 for apparent tachycardia with rates reported up to 240 beats/min on telemetry, and subjective complaint of chest discomfort. Telemetry revealed a wide-complex rhythm concerning monomorphic VT (Figures 1 and 2). Post–rapid-response electrocardiogram showed normal sinus rhythm with a QTc interval of 460 milliseconds (Figure 3). Intravenous magnesium (4 g) was administered, and QT-prolonging medications were held.

Despite alarming telemetry findings, bedside evaluation demonstrated no hemodynamic instability. The patient was alert, with a palpable pulse of 93 beats/min, blood pressure of 120/85 mm Hg, oxygen saturation of 97%, and normal respiratory rate. Cardiac examination was unremarkable. Laboratory testing revealed negative cardiac troponins, hypomagnesemia (1.6 mg/dL), and hypokalemia (3.0 mmol/L), which were corrected. Cardiology consultation was obtained.

**Figure 4:**
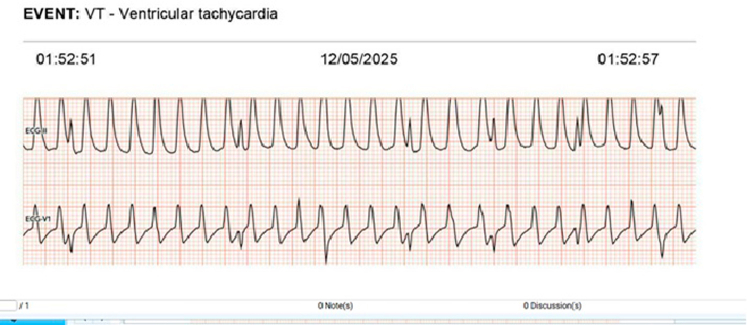
Telemetry Snippet With Normal QRS Complexes Seen Embedded in the Pseudo-Ventricular Tachycardia Tracing

Initial telemetry evaluation showed findings concerning for atrial flutter with 3:1 atrioventricular conduction with aberrancy. Careful clinical correlation revealed tremor-like movements occurring concurrently with the abnormal telemetry alarms and ECG findings. Detailed review of rhythm strips demonstrated preserved underlying QRS complexes embedded within a high-amplitude VT-like waveform (Figures 2 and 4), consistent with tremor-induced ECG artifact rather than true VT.

The patient was managed conservatively with electrolyte repletion and adjustment of psychiatric medications for tremors and anxiety. No further arrhythmic events occurred. She was discharged to a mental health facility subsequently. Recognition of telemetry artifact through careful bedside assessment and rhythm interpretation prevented unnecessary antiarrhythmic therapy and invasive cardiac evaluation.

## Discussion

ECG/telemetry artifact continues to remain a significant diagnostic challenge in hospitalized patients on continuous telemetry monitoring, as highlighted by Baranchuk et al,^6^ underscoring the importance of clinical correlation.^7^^,^^8^ The repetitive movements seen in tremors can generate rapid, high-amplitude tremor-induced artifacts that closely mimic cardiac arrhythmias, VT in our case, often triggering emergency responses and empirical antiarrhythmic therapy.^5^ These telemetry prompts should warrant immediate bedside assessment before an escalation of care is activated.

Several features can be used to help differentiate between actual VT and artifact. One of the key clinical manifestations is that the patient will still be hemodynamically stable even though they appear to be in extreme tachycardia.^9^ The discrepancy between the telemetry and actual patient heart rate may be proved by the examination at the bedside, including pulse palpation and cardiac auscultation. By closely looking at the rhythm strip, one can observe the existence of the QRS complexes concealed in the distortion of the baseline, the abnormal morphology at various leads, and temporal correlation with patient movement or tremors. If present, such features would lean more toward an artifact rather than a real ventricular arrhythmia. Furthermore, a study by Huan et al^10^ described certain signs to look for in patients with telemetry finding of VT-like appearance, describing “sinus sign,” “spike sign,” and “notch sign” as hallmarks of a diagnostic algorithm for differentiating pseudo-VT from VT on telemetry. Although most pseudo-VT can be differentiated from true VT by comparing multichannel telemetry tracings as described by Steinberg et al,^8^ our case describes similar appearance in all leads (Figures 1, 2, and 4), posing a diagnostic pitfall.

This patient's electrolyte disturbances, hypomagnesemia, and hypokalemia, present at baseline and seen after the rapid response, increased suspicion of a true VT, hence the cardiology consultation. These electrolyte abnormalities in our patient were likely secondary to poor oral intake in the setting of functional decline and possibly catatonia. Hypomagnesemia and hypokalemia are both known risk factors for ventricular arrhythmias.^6^ Although, these electrolyte abnormalities do not exacerbate tremors, the concurrence of these electrolyte abnormalities with VT-like appearance on telemetry poses a significant diagnostic pitfall.

This case shows that familiarity with the morphology of artifacts, close clinical examination, and algorithmic approach to artifacts can significantly reduce the number of unnecessary diagnostic tests and treatments. Most recent discoveries of tremor-associated artifact led to the conservative form of management, where emphasis was given to address the information on underlying causes, rather than advance to antiarrhythmic treatment or invasive intervention. Failure to recognize such artifacts may lead to avoidable and expensive unnecessary cardiac surgeries and/or interventions before the appropriate diagnosis is achieved, as demonstrated in previous research by Knight et al.^4^

ECG artifacts simulating VT have been adequately portrayed in the literature. Motion-related artifacts due to tremors, shivering, or Parkinsonian movements are among the most common causes of pseudo-VT.^6^^,^^8^ Preserved underlying QRS complexes and the absence of hemodynamic compromise are critical diagnostic clues. Despite increased awareness, artifact-related misdiagnosis continues to occur, underscoring the importance of bedside evaluation and careful rhythm interpretation. Our study will therefore add literature and value to the collective medicine practice to identify such artifacts and use bedside examination and temporal correlation before moving to escalated care.

## Conclusions

Tremors can lead to an ECG artifact closely mimicking VT and lead to inappropriate escalation of care. This case highlights the diagnostic pitfalls of overreliance on telemetry without bedside correlation and underscores the importance of integrating clinical stability, physical examination, and detailed rhythm analysis to avoid unnecessary interventions.

## Funding Support and Author Disclosures

The authors have reported that they have no relationships relevant to the contents of this paper to disclose.
